# An *in silico* Approach Reveals Associations between Genetic and Epigenetic Factors within Regulatory Elements in B Cells from Primary Sjögren’s Syndrome Patients

**DOI:** 10.3389/fimmu.2015.00437

**Published:** 2015-08-26

**Authors:** Orsia D. Konsta, Christelle Le Dantec, Amandine Charras, Wesley H. Brooks, Marina I. Arleevskaya, Anne Bordron, Yves Renaudineau

**Affiliations:** ^1^INSERM ESPRI, ERI29/EA2216, SFR ScInBioS, LabEx IGO “Immunotherapy Graft Oncology”, Innovative Medicines Initiative PRECISESADS, Réseau épigénétique et réseau canaux ioniques du Cancéropole Grand Ouest, European University of Brittany, Brest, France; ^2^Department of Chemistry, University of South Florida, Tampa, FL, USA; ^3^Department of Rheumatology, Kazan State Medical Academy, Kazan, Russia; ^4^Laboratory of Immunology and Immunotherapy, CHU Morvan, Brest, France

**Keywords:** Sjögren’s syndrome, genetics, epigenetics, polymorphism, *in silico*, histone modifications, B cells

## Abstract

Recent advances in genetics have highlighted several regions and candidate genes associated with primary Sjögren’s syndrome (SS), a systemic autoimmune epithelitis that combines exocrine gland dysfunctions, and focal lymphocytic infiltrations. In addition to genetic factors, it is now clear that epigenetic deregulations are present during SS and restricted to specific cell type subsets, such as lymphocytes and salivary gland epithelial cells. In this study, 72 single nucleotide polymorphisms (SNPs) associated with 43 SS gene risk factors were selected from publicly available and peer reviewed literature for further *in silico* analysis. SS risk variant location was tested revealing a broad distribution in coding sequences (5.6%), intronic sequences (55.6%), upstream/downstream genic regions (30.5%), and intergenic regions (8.3%). Moreover, a significant enrichment of regulatory motifs (promoter, enhancer, insulator, DNAse peak, and expression quantitative trait loci) characterizes SS risk variants (94.4%). Next, screening SNPs in high linkage disequilibrium (*r*^2^ ≥ 0.8 in Caucasians) revealed 645 new variants including 5 SNPs with missense mutations, and indicated an enrichment of transcriptionally active motifs according to the cell type (B cells > monocytes > T cells ≫ A549). Finally, we looked at SS risk variants for histone markers in B cells (GM12878), monocytes (CD14^+^) and epithelial cells (A548). Active histone markers were associated with SS risk variants at both promoters and enhancers in B cells, and within enhancers in monocytes. In conclusion and based on the obtained *in silico* results that need further confirmation, associations were observed between SS genetic risk factors and epigenetic factors and these associations predominate in B cells, such as those observed at the FAM167A–BLK locus.

## Introduction

Primary Sjögren’s syndrome (SS) is a systemic autoimmune epithelitis affecting exocrine glands, such as salivary and lacrimal glands (1). The clinical manifestations of SS include dry mouth (xerostomia), dry eyes (keratoconjunctivitis sicca), systemic features, and patients have a 20- to 40-fold increased risk of developing lymphoma (2–4). Histological examination shows focal and peri-epithelial T and B cell infiltration plus macrophages in exocrine glands and parenchymal organs, such as kidney, lung, and liver (5). SS is characterized by the presence of circulating autoantibodies (Ab) against the sicca syndrome (SS)A/Ro and SSB/La ribonucleoprotein particles (6).

It is estimated that there are over 120 million single nucleotide polymorphisms (SNPs) in the human genome (NCBI dbSNP database, Build 143) and, among them, hundreds are disease risk variants for autoimmune diseases (AID) with the particularity that they are for the vast majority excluded from protein-coding regions (exon) and present within regulatory areas (7, 8). Regulatory SNPs control genes through an effect on (i) the transcriptional machinery when present within a gene regulatory region [promoter, enhancer, insulator (a gene regulatory element that blocks interaction between enhancers and promoters), and expression quantitative trait loci (eQTL)], (ii) the spliceosomal complex formation that controls intron excision, (iii) the activation of mRNA non-sense-mediated decay (NMD), and (iv) the control of messenger RNA stability through microRNA (3′-UTR). In SS, the list of genetic variations is growing with the particularity that the odds ratio (OR) is usually modest (OR < 1.5) with the exception of the HLA genes that have a significant OR (usually >2) (9). The associated risk genes analysis supports immunopathological pathways in SS, such as antigen presentation, cytokine signaling, and the NF-κB pathway (10). The characterization of regulatory SNPs in SS remains to be established.

In SS, several arguments support a role for epigenetic deregulation in disease initiation and progression (11, 12). The first clue was that two drugs, procainamide and hydralazine, induced SS in humans by blocking DNA methylation (13). Moreover, defects in DNA methylation characterize T cells, B cells, and salivary gland epithelial cells from SS patients (14–16), and such defects were associated with the expression of genes usually repressed by DNA methylation, such as transposons and miRNAs in salivary glands from SS patients (17, 18). Last, but not least, histone epigenetic markers and ribonucleoprotein post-translational modifications are immunogenic leading to autoAb production (14).

Accordingly, the aim of this work was to test the association between genetic and epigenetic determinants in SS. In the following, we pursue a two-staged analysis. First, we characterized a large panel of SS risk variants to reveal that they are predominantly present within regulatory elements. Second, we further explored the striking associations of those regulatory elements with cellular specificity and particularly in immune cells.

## Materials and Methods

### SS genetic risk factors

Data mining based on peer reviewed literature information (PubMed) and publicly available databases (centralgwas.org) served in the compilation of a list of 43 gene risk factors and their reported variants (*n* = 72) in SS (Table S1 in Supplementary Material) (19–49). The number of SS patients and controls were also reported as well as the OR average (95%), when available. The gene list used in this study was manually updated further to include gene function, SNP number (dbSNP database), and genomic location according to the human genome reference GRCh38. Genetic variants and their observed associations with clinical and functional phenotype were submitted to The National Center for Biotechnology Information (NCBI) ClinVar database^1^. The gene list was tested with the FatiGO web interface AmiGO2^2^ for functional enrichment.

### Functional/regulatory genome annotation data

The variant effect predictor (VEP) tool^3^ was used to determine the location of the variants (exon, intron, 5′/3′-UTR, Up/Downstream genic sequence, and intergenic section) and their consequences [missense, non-coding transcript, splice donor variant, and target of non-sense-mediated mRNA decay (NMD)].

Assessment of the SNPs functional relevance was further completed by requesting Regulome DB V1.1^4^, and HaploRegV2^5^, data bases (50, 51) for promoter [RNA polymerase II (Pol II) binding], enhancer (H3K27Ac, H3K36me3, and/or H3K27me3 binding in the absence of Pol II binding), insulator [CCCCTC-binding factor (CTCF)], transcription factor (TF) binding, DNase peak, and eQTL.

### Linkage disequilibrium

Following SNP selection, the HaploRegV2 web portal was used to identify SNPs in linkage disequilibrium (LD, *R*^2^ ≥ 0.80) in Europeans from the 1000 genome project using a maximum distance between variants of 200 kb in order to cover the enhancer elements (51).

### Statistical analysis

Pearson’s Chi-squared test with Yate’s continuity correction, when appropriate, was used to evaluate the significance of differences between the regulatory motifs and the histone chromatin immunoprecipitation (ChIP) experiments. A probability (*P*) of <0.05 was considered significant.

## Results

### Autoimmune-related genes associated with SS

**Figure 1:**
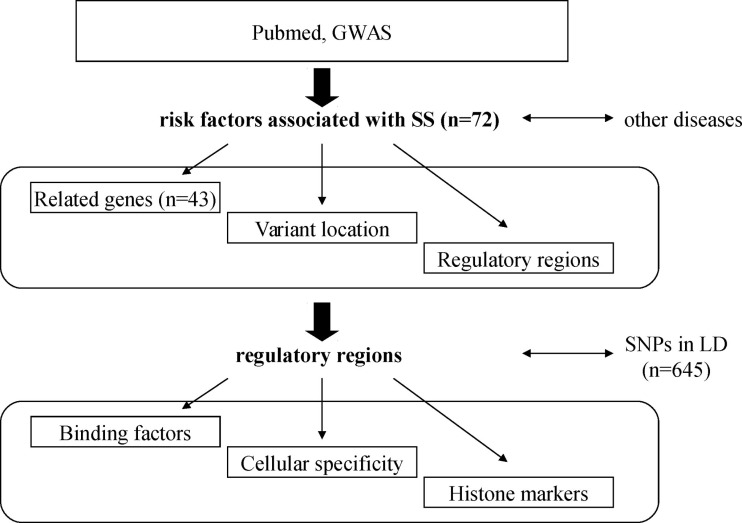
**Schematic diagram representing the analysis strategy**. SS-associated risk variants were obtained from peer reviewed scientific literature, and their location determined. Next, they were tested for the enrichment of binding factors and cellular specificity. Presence of single nucleotide polymorphisms (SNP) in high linkage disequilibrium (LD) was also assessed.

A list of 43 SS-associated gene risk factors corresponding to 72 SNPs, referred to as SS risk variants, was extracted from the scientific literature (Figure 1). Among the risk factors, half (36/72) were associated with another AID (systemic lupus erythematosus, rheumatoid arthritis, systemic sclerosis, inflammatory bowel disease, autoimmune thyroiditis disease, insulin-dependent diabetes, primary biliary cirrhosis, autoimmune hepatitis), allergy, infections, and cancer, including B/T cell lymphomas. This partial overlap suggests that both common and distinct genetic traits are present in SS and equally distributed.

### Variant location

**Figure 2:**
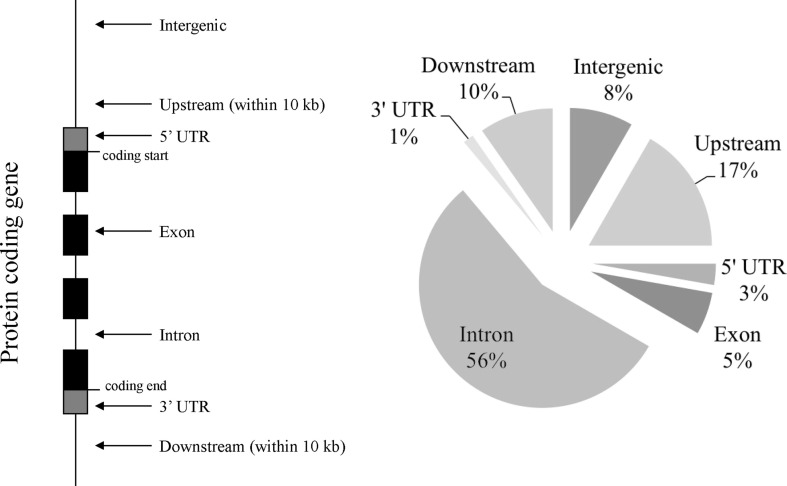
**Occurrences of SS risk variants according to the protein-coding gene location**.

Next, we used the VEP predictor tool in order to test the location of the different variants (Figure 2). Applied to the 72 SS risk variants, the VEP tools identified 4/72 (5.6%) exonic variants with missense mutations (IL17F, rs763780; MBL2, rs1800450; PTPN22, rs2476601; and TNFA1P3, rs2230926), and 40/72 (55.6%) intronic variants including an alternative splice donor variant (IRF5, rs2004640) and 4 variants that were predicted as targets of non-sense-mediated mRNA decay (NMD: ICA1, rs17143355; SLC25A40, rs10276819; STAT1, rs13005843; and TNIP1, rs6579837). Moreover, two 5′-UTR variants (CD14, rs2569190; and NCR3, rs11575837), one 3′-UTR variant (IL10, rs3024498), 12/72 (16.7%) upstream genic variants, 7/72 (9.7%) downstream genic variants, and 6/72 (8.3%) intergenic variants (>10 kb) were also observed.

### Regulatory regions and DNA binding molecules

**Figure 3:**
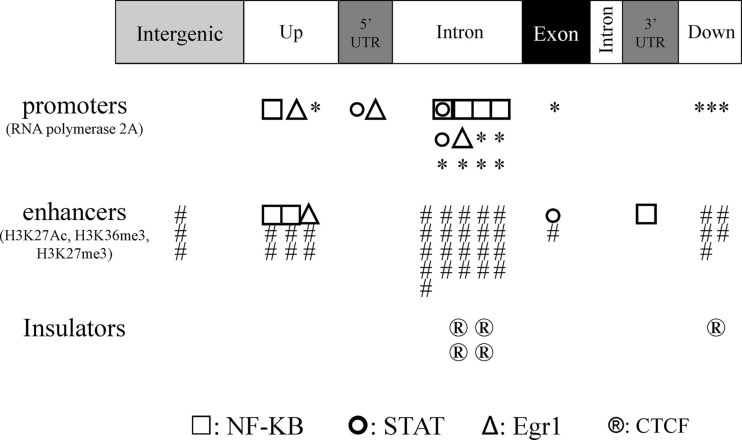
**Regulatory SS risk variants were subdivided into three groups: promoters (polymerase II binding), enhancers (H3k27Ac, H3k36me3, and/or H3k7me3 binding), and insulators (CTCF binding)**. Regulatory SS risk variants were further located according to the protein-coding gene, and the predominant transcription factor associated with each SS risk variant is indicated (NF-κB, STATs, and Egr1).

We then used a combination of three tools based on information from the ENCODE program (VEP) and from both the ENCODE and the Roadmap Epigenome programs (RegulomDB and HaploReg v2) to determine whether SS risk variants are likely to be within promoters, enhancers, or insulators. These regulatory motifs were defined according to the available ChIP results from multi-cell analysis showing 21/72 (29.2%) promoters, 41/72 (56.9%) enhancers, and 5/72 (6.9%) insulators. Of particular note, within the four SNPs with missense mutations, one promoter and two insulators were detected (Figure 3). Moreover, 34/72 (47.2%) DNase hypersensitive regions (DNase peak) and 12/72 (16.7%) eQTL were recovered. Looking specifically at promoters and enhancers, data from ChIP experiments revealed that NF-κB (*n* = 5), STATs (*n* = 3), and EGR-1 (*n* = 3) were predominant in promoters, and NF-κB (*n* = 3) in enhancers. For the remaining 5/72 (6.9%) SNPs, no regulatory functions were assigned which is significantly lower than the expected rate of 56.2% (*P* < 10^−6^) (50).

### Genes in high linkage disequilibrium

In order to improve the analysis, we used the HaploReg v2 tool to include 645 new SNPs that were identified to be in high LD with the 72 annotated SNPs (Table 1). This tool identifies 34 new genes, including one microRNA (Mir4752), five SNPs with missense mutations (rs1041981 in LTA; rs78957773 in MCCD1, rs2230539 in PKN1, and rs52817781 plus rs2233290 in TNIP1), and indicated an overall 15.8-fold enrichment of enhancers in human embryonic cells (H1; *P* = 0.0001) and 4.9-fold enrichment of enhancers in the GM12878 EBV-transformed lymphoblastoid B cell line (*P* = 0.0014). A significant enrichment of transcriptionally active sites, identified by DNAse enrichment analysis, was observed in the SNP set with a 21-fold increase in GM12878 lymphoblastoid B cells (*P* < 10^−6^), 10.8-fold increase in CD14^+^ monocytes (*P* = 0.00002), 9.2-fold increase in CD20^+^ B cells (*P* = 0.001), 8.2- to 8.5-fold increase in T cells (*P* < 0.005), and 5.7-fold increase in the A549 epithelial cell line (*P* = 0.005). Moreover, gene ontology biological process analysis (AmiGO2) identified “regulation of immune response” (*P* = 2.16 × 10^−18^ and 9.2 × 10^−17^), “positive regulation of cytokine production” (*P* = 1.56 × 10^−11^ and 1.22 × 10^−13^), “response to molecule of bacterial origin” (*P* = 4.9 × 10^−7^ and 6.2 × 10^−9^), and “cellular response to interferon” (*P* = 1.82 × 10^−7^ and 1.86 × 10^−7^) as the most significant functions associated with the SS gene risk factors tested alone or associated with the genes revealed by the LD analysis, respectively.

**Table 1:** **Gene risk factors associated with primary Sjögren’s syndrome (SS)**.

**SS gene risk factors extracted from the literature**
BAK1, BCL2, BLK, C4A, CCL2, CD14, CD40, CD40LG, CHRM3, CXCR5, EBF1, FAM167A, GTF2I, HLA DPB1, HLA-DQA1, HLA-DQB1, HLA-DRA, ICA1, IKBKE, IL10, IL12A, **IL17F**, IL21, IRF5, LILRA3, LTA*, **MBL2**, NCR3, NFKB1, OR2B11, PKN1*, **PTPN22**, SLC25A40, STAT1, STAT4, TNF, **TNFA1P3**, TNFSF13B, TNIP1*, TNPO3, TNFSF4, Trim21

**New genes in high linkage disequilibrium**

ABCB1, AC053545.3, ADAD1, AF213884.2, APBB3, ATP6V1G2, ATP6V1G2-DDX39B, BTNL2, C6orf10, CCL11, CCL7, CTD-2049J23.2, DBF4, GGNBP1, HCG23, HLA-DPA1, IL2, KIAA1109, MCCD1*, Metazoa_SRP, MIR4752, NDUFA2, NFKBIL1, PTGER1, RP11-10J5.1, RP11-356I2.2, RP11-356I2.4, RP3-527F8.2, RP5-998H6.2, RSBN1, RUNDC3B, SLC25A40, snoU13, XXbac-BPG254F23.7

*Bold missense mutations*.

*Missense mutations detected by linkage disequilibrium.

### Cell type-specific analysis revealed activated enhancer and promoter histone markers at SS risk variants in B cells

To further explore cell type specific activation in promoters and enhancers at SS risk variants and according to the critical role played in the disease by epithelial cells, lymphocytes, and macrophages, we selected from the 18 ENCODE available cells: the human lung adenocarcinoma cell line A549 for epithelial cells, the GM12878 lymphoblastoid cells for B cells, and the peripheral blood CD14^+^ monocytes for macrophages. For these three cell types, we mapped SS risk variants to markers of active promoters (H3K4me2, H3K4me3, and H3K9Ac), and to markers of active enhancers (H3K36me3 and H3K4me1) (52). In addition, H3K27Ac was selected as a marker of activity, and H3K27me3 as an inactive marker of enhancers.

**Figure 4:**
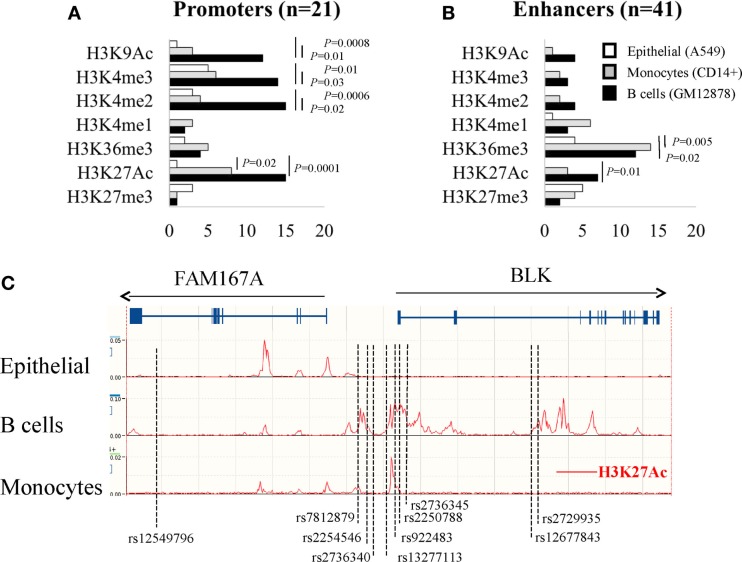
**Analysis of histone modifications in the promoters (A) and enhancers (B) of SS risk variants within A549 epithelial cells, B cell lymphobastoid GM12878 cells, and CD14^+^ monocytes**. **(C)** In the lymphoblastoid GM12878 B cell line, SS genetic variants and the active histone markers H3k27Ac are co-localized in and around the FAM167A–BLK locus (http://www.ensembl.org).

As shown in Figure 4A and with regards to the 21 promoter SS risk variants, the three active promoter markers (H3K4me2, H3K4me3, and H3K9Ac) were significantly enriched in B cells (GM12878) in contrast to the epithelial cells (A549) and monocytes (0.01 < *P* < 0.0006, Chi square with Yate’s correction). The active marker H3K27Ac was enriched in B cells and monocytes in contrast to epithelial cells (*P* = 0.0001 and *P* = 0.02, respectively).

The same analysis was performed with the 41 enhancer SS risk variants (Figure 4B) revealing an enrichment of the enhancer active marker H3K36me3 in both B cells and monocytes in contrast to A459 cells (*P* = 0.02 and *P* = 0.005, respectively). The active marker H3K27Ac was enriched in B cells (*P* = 0.0001), and, although not significant, there is a trend for a monocyte enrichment in contrast to epithelial cells. In summary, these findings highlight the critical role of epigenetic factors in B cells to control both promoter and enhancer SS risk variants, and in monocytes to control enhancer SS risk variants.

### FAM167A–BLK locus

In order to validate our observations, and based on three reports, including the genome wide association study (GWAS) performed by Lessard et al., in 395 patients with SS and 1975 controls from European origins (31, 35, 41), the FAM167A–BLK locus (Chr 8:11421463-11564604) was selected to position the 8 FAM167A–BLK SS risk variants plus two 5′-UTR variants selected from the LD analysis and previously identified as lupus risk variants (53). These two SNPs are in high LD with 4/8 SS risk variants [rs922483 is in high LD with rs2736340 (*r*^2^ = 0.81), rs13277113 (*r*^2^ = 0.83), and rs2736345 (*r*^2^ = 0.96); and rs2250788 is in high LD with rs2254546 (*r*^2^ = 0.98)]. As shown in Figure 4C, the 10 selected SNPs were positioned in the FAM167A–BLK locus revealing three groups. The first group contains an isolated SNP (rs12549796) that was present in an intronic part of the FAM167A gene. A second group (*n* = 7) was present in the vicinity of the BLK promoter and exon 1, and a third group (*n* = 2) was present ~35 kb downstream BLK promoter.

Next, as revealed by querying the Ensembl database using H3K27Ac to mark active promoters and enhancers, SNPs were positioned within 9/10 H3K27Ac active motifs in B cells (GM12878), which is in contrast to 2/10 H3K27Ac active motifs in monocytes, and none in epithelial cells. Such associations between genetic and epigenetic factors within regulatory elements in B cells for the FAM167A–BLK locus were further reinforced by using the RegulomeDB tool that summarizes results from the ENCODE and Epigenetic Roadmap programs. As indicated Table 2, the RegulomeDB tool supports that SS risk factors at FAM167A–BLK locus would predominantly affect B cells (lymphoblastoid and naive B cells) and, to a lesser extent, monocytes, T cells (naïve, TH2, and Treg), mesenchymal stem cells, and fibroblasts.

**Table 2:** **Summary of the cell type enrichment markers for FAM167-BLK variants according to the RegulomeDB prediction web tool**.

SNP	Promoter (histone markers)	Enhancer (histone markers)	Open chromatin (DNase)	eQTL	Pol II (ChIP-Seq)
rs12549796	No	Mesenchymal stem cells	Fibroblast	No	No
rs7812879	No	B lymphoblastoid, B cells, monocytes	No	B lymphoblastoid	No
rs2254546	No	B cells	No	B lymphoblastoid	No
rs2736340	No	B cells	No	B lymphoblastoid, monocytes	No
rs13277113	B lymphoblastoid	B cells, monocytes	B lymphoblastoid, B cells	B lymphoblastoid	No
rs922483	B lymphoblastoid, B cells, T cells, monocytes	Fibroblast	B lymphoblastoid, B cells, TH2, Treg	B lymphoblastoid	B lymphoblastoid
rs2250788	B lymphoblastoid, B cells, T cells, monocytes	Fibroblast	B lymphoblastoid, B cells	No	B lymphoblastoid
rs2736345	B lymphoblastoid, B cells, T cells	Fibroblast, Mesanchymal stem cells	B lymphoblastoid, B cells, TH2	B lymphoblastoid	B lymphoblastoid
rs12677843	No	No	No	No	No
rs2729935	No	B lymphoblastoid, B cells	No	No	No

## Discussion

Primary SS is an autoimmune disease with a genetic basis in which at least 40 gene risk factors may be involved, including BLK, IRF5, STAT4, and the HLA locus. However, these genetic risk factors alone cannot explain all of the disease risk factors and, in particular, environmental risk factors (e.g., viruses, hormones …) that are likely to play a critical role in the process of the disease. Given the complexity of the disease, epigenetic analyses are conducted to provide new insights into the disease as DNA methylation patterns, chromatin structures, and microRNA are influenced both by the genetic machinery and by environmental factors (13, 54, 55). The primary role of the epigenome is to regulate, in a cell-specific manner, cellular development, and differentiation and such effects vary between individuals with age as revealed by testing identical twins (56), or between smokers and non-smokers (57). Furthermore, genetic variants and, in particular, non-coding and regulatory SNPs can influence cell type specific regions marked by accessible regions, thus opening new perspectives to better characterize disease risk factors and cell types contributing to the diseases which was the aim of the present *in silico* analysis.

Applied to SS, such strategy was fruitful in suggesting the existence of associations between genetic and epigenetic alterations in the setting of the disease. Indeed, a cell-specific overlap exists between identified SS risk variants and the regulatory switches found by the ENCODE program, thus suggesting that DNA–protein binding and gene transcription are affected by the SNPs. Remarkably, almost all SS risk variants tested (94.4%) had *in silico* evidence of regulatory functions including the 3/4 missense SNPs and the 37/40 intronic SNPs. In addition and according to our *in silico* observations that need further confirmation, it could be postulated that SS risk variants control DNA-protein binding leading to the regulation of cell-specific promoters (Pol II, NF-κB, STATs), enhancers (NF-κB), and insulators (CTCF). These results also suggest that there is an effect on some common pathways (NF-κB, STATs) previously described to be affected in SS (10).

The genetic and epigenetic fine mapping of autoimmune risk factors was recently performed in 21 AID with the notable exception of SS (7). In line with our observations, it was observed that autoimmune risk variants were mostly non-coding (90%) and map predominantly to H3K27Ac positive immune-cell enhancers (60%) and promoters (8%). Next, a T cell signature was observed in nearly all of the AID tested except in lupus and primary billiary cirrhosis (two AID frequently associated with SS) that present a B cell signature, and type I diabetes with pancreatic islets. Finally, it was reported that autoimmune risk factors were enriched within binding sites for immune-related TFs, such as Pu-1 and NF-κB. As a consequence, the physiopathology of AID needs to be updated according to the recent progress in epigenetics (54).

Some limitations are inherent in this type of study. First, cells used in the ENCODE program are predominantly cell lines that are different from primary cells, such as the lymphoblastoid GM12878 B cell line, that results from EBV transformation of peripheral blood mononuclear cell using phytohemagglutinin as a mitogen. New results using primary cells, which are available from the Epigenome Roadmap program further supports similarities between lymphoblastoid GM12878 B cells and purified human CD20^+^ B cells as we observed for the FAM167A–BLK locus when using the RegulomeDB tool. Second, although the ENCODE program is an extensive resource; the program is limited to certain cell types and DNA binding elements that limit the interpretation. Third, many SNPs are in tight genetic linkage and, as a consequence, genetic risk variants may not be causal, but rather reveal the presence of a linked SNP that is functionally relevant to the pathogenesis. Such a situation may be suspected for different SNPs tested from our selection since the LD analysis has revealed new missense mutations as well as new gene risk factors that need to be tested, such as chemokines (CCL7 and CCL11), cytokines (IL2) and the miRNA4752. Two SNPs in CCL11 have been associated with germinal center-like structure formation in SS patients (47), and CCL11 (Eotaxin) circulating levels were reduced in SS patients (58).

While the function of the protein encoded by FAM167A is unknown, the tyrosine kinase BLK controls B cell development and is activated after B cell receptor engagement. The FAM167A–BLK locus is associated with several AID, such as SS, lupus, rheumatoid arthritis, scleroderma, and vasculitis. Among them, two risk alleles (rs132771113 and rs9222483) are known to control BLK transcription during B cell development (53, 59). Moreover, by integrating epigenetic fine mapping, we further observed that all BLK-associated SS risk variants, including the two previously described, were all present within epigenetic marks in B cells. Altogether, this example illustrates the value of integrating epigenetic resources for investigating the complex mechanisms by which non-coding risk variants could modulate gene expression.

Last but not least, the B cell subset identified from our *in silico* study deserves several comments. First, B cell qualitative abnormalities have been reported in SS with important perturbations in peripheral blood B cell profiling and B cell migration within exocrine glands (5, 60). Second, the association between the incidences of B cells in salivary gland epithelial cells has been addressed as well as the formation of ectopic germinal centers and transformation to B cell lymphoma (61). Third, non-HLA genetic associations in SS are predominantly related to B cell genes (BTK, CD40, EBF-1 …) as we observed in our selection. Fourth, a recent study reported DNA methylation changes in B cells and such changes predominate within loci containing SS risk factors (16). Altogether, these observations provide rationale for targeting B cells in SS along with the observations that depleting B cells with Rituximab or targeting BAFF with Belimumab are both effective (62, 63).

In conclusion, we have tested, as a proof of concept, a novel approach that integrates both epigenetic information and results from genomic analysis to further enhance the value of the genetic risk factors highlighted in complex diseases, such as SS. Future work needs to be done in order to confirm experimentally the cellular specificity and the functional role of the characterized regulatory SNPs. Another consequence is that such approach could be used to select and/or propose future therapeutic drugs in SS as epigenetic mechanisms are reversible.

## Conflict of Interest Statement

The authors declare that the research was conducted in the absence of any commercial or financial relationships that could be construed as a potential conflict of interest.

## Supplementary Material

The Supplementary Material for this article can be found online at http://journal.frontiersin.org/article/10.3389/fimmu.2015.00437

## Footnotes

^1^http://www.ncbi.nlm.nih.gov/clinvar

^2^http://amigo2.geneontology.org

^3^http://www.ensembl.org

^4^http://regulome.stanford.edu

^5^http://www.broadinstitute.org
