# Supplementary material for: An in silico Approach Reveals Associations between Genetic and Epigenetic Factors within Regulatory Elements in B Cells from Primary Sjögren’s Syndrome Patients
Source: Front Immunol. 2015 Aug 26;6:437. doi: 10.3389/fimmu.2015.00437 (PMC4549647; doi:10.3389/fimmu.2015.00437)
Supplement: Supplementary file 1 [file Table_1.PDF]

Supplementary table 1: Genetic risk factors associated with Sjögren's syndrome (see material and methods section for more details).

Gene	Function	Locus	SNP	Genomic location (GRCh38)	Annotation (VEP tool)	Functional relevance	ENCODE & Roadmap projects	Average OR (95% CI)	Patients / controls tested	Disease specificity	Reference
BAK1	Bcl2 antagonist	6p21.31	rs513349	6:33573942	Intron	Enhancer + Insulator (CTCF)	TF + DNase peak + Histone markers	0.51 (0.36-0.72)	99/252	SLE	(Delgado-Vega *et al.*, 2010)
BAK1	Bcl2 antagonist	6p21.31	rs5745582	6:33578721	Intron	Promoter (Pol2) + Insulator (CTCF)	eQTL + TF binding	1.56 (1.09-2.25)	99/252	-	(Delgado-Vega *et al.*, 2010)
BCL2	Apoptosis regulator	18q21.33	rs7230970	18:63153843	Intron	Promoter (Pol2)	TF + Tbp motif	0.78 (0.65-0.93)	540/532	-	(Nordmark *et al.*, 2011)
C4A	Complement	6p21.33	rs396458	6:32037872	Downstream	Enhancer	Histone markers	0.58 (0.39-0.86)	540	-	(Bolstad *et al.*, 2012)
CCL2	Chemokine	17q12	rs1024611	17:34252769	Upstream	Promoter (Pol2, NFKB1, PU1)	TF + DNase peak/footprint + IRF2	0.48 (0.23-1.00)	52/164	RA, IBD, DID, infection, cancer	(Iwamoto *et al.*, 2010)
CD14	Monocyte differentiation	5q31.3	rs2569190	5:140633331	5' UTR	Promoter (STAT3, Jun)	eQTL + TF + DNase peak/footprint	1.32 (1.11-1.57)	540/532	IBD, Allergy	(Nordmark *et al.*, 2011)
CD40	TNF receptor 5	20q13.12	rs1883833	20:46136819	Intergenic	Unknown	No	0.75 (0.63-0.89)	540/532	-	(Nordmark *et al.*, 2011)
CD40LG	CD40 ligand	Xq26.3	rs3092949	X:136645176	Upstream	Enhancer	Histone markers	0.77 (0.64-0.93)	540/532	-	(Nordmark *et al.*, 2011)
CHRM3	Muscarinic receptor	1q41	rs7548522	1:239835325	Intron	Enhancer	Histone markers	1.93 (1.24-3.01	530/532	-	(Appel *et al.*, 2011)
CXCR5	Chemokine	11q23.3	rs7119038	11:118867572	Intergenic	Enhancer (H3K27me3)	Histone mark	0.74 (0.64-0.86)	395/1975	-	(Lessard *et al.*, 2013)
EBF1	EarLyB B-cell factor	5q33.3	rs3843489	5:158753215	Intron	Enhancer (Pax5)	TF	1.68 (1.29-2.18)	540/532	-	(Nordmark *et al.*, 2011)
EBF1	EarLyB B-cell factor	5q33.3	rs869593	5:159003867	Intron	Enhancer (Fos, Myc)	TF + DNase peak	-	540/532	-	(Nordmark *et al.*, 2011)
FAM167A-BLK	B cell kinase	8p23.1	rs12549796	8:11432188	Intron	Insulator (CTCF)	DNase peak	1.37 (1.15-1.63)	540/532	-	(Nordmark *et al.*, 2011)
FAM167A-BLK	B cell kinase	8p23.1	rs7812879	8:11482672	Intergenic	Enhancer	eQTL	0.86 (0.71-1.06)	555/597	SLE	(Sun *et al.*, 2013a)
FAM167A-BLK	B cell kinase	8p23.1	rs2254546	8:11486171	Intergenic	Enhancer	eQTL+NFAT binding	0.85 (0.69-1.04)	555/597	SLE, Kawasaki	(Sun *et al.*, 2013a)
FAM167A-BLK	B cell kinase	8p23.1	rs2736340	8:11486464	Upstream	Enhancer	eQTL + TF + Histone markers	-	540/577	SLE, PR, SSc, Kawasaki	(Sun *et al.*, 2013b)
FAM167A-BLK	B cell kinase	8p23.1	rs13277113	8:11491677	Upstream	Promoter (Pol2, MEF2A)	eQTL + TF binding + DNase peak		540/577	SLE, RA,SSc	(Sun *et al.*, 2013b)
FAM167A-BLK	B cell kinase	8p23.1	rs2736345	8:11494976	Intron	Promoter (Pol2, NFKB1)	eQTL + TF + DNase peak	1.30 (1.16-1.47)	395/1975	-	(Lessard *et al.*, 2013)

FAM167A-BLK	B cell kinase	8p23.1	rs12677843	8:11529680	Intron	Enhancer	Histone markers		540/532	-	(Nordmark *et al.*, 2011)
FAM167A-BLK	B cell kinase	8p23.1	rs2729935	8:11532274	Intron	Enhancer	Histone markers	1.30 (1.16-1.46)	395/1975	-	(Lessard *et al.*, 2013)
GTF2I	Transcription factor	7q 11.3	rs117026326	7:74711703	Intron	Enhancer	histone markers, MECP2 motif	2.20 (1.99-2.43)<br>1.70 (1.01-2.88)	542/1050<br>72/196	-	(Li *et al.*, 2013)<br>(Zheng *et al.*, 2015)
HLA DPB1	Ag presentation	6p21.32	rs4282438	6:33104395	Intron	Enhancer	Histone markers	1.58 (1.45-1.72)	542/1050	cancer	(Li *et al.*, 2013)
HLA DQA1	Ag presentation	6p21.32	rs9271588	6:32623176	Intron	Promoter (Pol2, NFKB1, OCT2, PU1, MEF2A)	eQTL + TF + DNase peak/footprint	0.57 (0.53-0.63)<br>0.41 (0.36-0.46)	542/1050<br>395/1975	-	(Li *et al.*, 2013)<br>(Lessard *et al.*, 2013)
HLA-DQA1	Ag presentation	6p21.32	rs116232857	6:32629287	Intron	Enhancer	Histone mark	3.53 (3.03-4.11)	395/1975	-	(Lessard *et al.*, 2013)
HLA-DQA1	Ag presentation	6p21.32	rs2187668	6:32638107	Intron	Promoter (Pol2)	eQTL + TF + DNase peak	2.47	574/451	SLE, CD, AIH, DID, RA, SSc, IBD	(Rusakiewicz *et al.*, 2013)
HLA-DQB1	Ag presentation	6p21.32	rs3129716	6:32689659	Intron	Promoter (Pol2, NFKB1, PAX5, OCT2)	eQTL + TF	3.45 (2.97-4.00)	395/1975	-	(Lessard *et al.*, 2013)
HLA-DRA	Ag presentation	6p21.32	rs3135394	6:32440720	Intron	Promoter (Pol2, STAT5)	TF	3.52 (3.02-4.10)	395/1975	-	(Lessard *et al.*, 2013)
HLA-DRA	Ag presentation	6p21.32	rs112357081	6:32451499	Intergenic (insertion)	Unknown	-	2.89 (2.43-3.44)	395/1975	-	(Lessard *et al.*, 2013)
ICA1	Islet cell autoantigen	7p21.3	rs17143355	7:8119939	Intron	target of NMD	No	0.66 (0.51-0.85)	540/532	-	(Nordmark *et al.*, 2011)
IKBKE	NF-KB pathway	1q32.1	rs17433804		Intron	Enhancer	TF + DNase peak		76/244*		(Reksten *et al.*, 2014)
IKBKE	NF-KB pathway	1q32.1	rs11117909	1:206476609	Intron	Enhancer	DNase peak + Histone mark	0.63 (0.47-0.84)	540/532	-	(Nordmark *et al.*, 2011)
IL10	cytokine	1q32.1	rs3024498	1:206768134	3’ UTR	Enhancer (NFKB1)	TF + DNase peak	0.76 (0.62-0.94)	540/532	cancer, infections, lymphoma B	(Nordmark *et al.*, 2011)
IL12A	cytokine	3q25.33	rs485497	3:160001345	Intron	Enhancer	DNase peak	1.29 (1.16-1.46)	395/1975		(Lessard *et al.*, 2013)
IL17F	cytokine	6p12.2	rs763780	6:52236941	Exon	missense	No		22	RA, SSc, IBD, GD, AITD, ITP, VKH, allergy, infections, cancer	(Paradowska-Gorycka *et al.*, 2014)
IL21	cytokine	4q27	rs6822844	4:122588266	Downstream	Enhancer	Histone markers	0.46 (0.23-0.95)	94/368	SLE, RA, DID, CD	(Maiti *et al.*, 2010)
IRF5	Transcription factor	7q32.1	rs3757387	7:128936032	Upstream	Enhancer	WRNIP1 binding	1.44 (1.29-1.62)	395/1975	-	(Lessard *et al.*, 2013)
IRF5	Transcription factor	7q32.1	rs2004640	7:128938247	Intron	Promoter (Pol2, E2F, EGR1) + Splice donor variant	TF + DNase peak	1.93 (1.15-3.42)	212/162	-	(Miceli-Richard *et al.*, 2007)
IRF5	Transcription factor	7q32.1	rs10488631	7:128954129	Downstream	Enhancer (GATA2)	TF + DNase peak	1.57 (1.23-1.99)	368/711	SSc, RA, SLE	(Nordmark *et al.*, 2009)

LILRA3	Receptor	19q13.4	rs103294	19:54293995	Downstream	Enhancer	eQTL + Histone markers	2.32 (1.38-3.88)	403/2169	SLE, RA, MS, cancer	(Du *et al.*, 2014)
LTA	Cytokine	6p21.33	rs909253	6:31572536	Intron	Promoter (Pol2, NFKB1, STAT5A)	TF + DNase peak	1.59 (1.34-1.89)	527/532	Myocardial infarction, psoriatic arthritis	(Bolstad *et al.*, 2012)
MBL2	Complement	10q21.1	rs1800450	10:52771475	Exon	Enhancer (p300) + Misense	TF + Histone markers	0.69 (0.54-0.92)	280/515	RA, infection	(Song *et al.*, 2014)
NCR3	Receptor	6p21.32	rs11575837	6:31592893	5' UTR	Promoter (Pol2, Egr1)	TF + DNase peak	0.38	574/451	Infection	(Rusakiewicz *et al.*, 2013)
NCR3	Receptor	6p21.32	rs2736191	6:31593133	Upstream	Promoter (Pol2, Egr1)	TF + DNase peak	0.43	574/451	infection	(Rusakiewicz *et al.*, 2013)
NFKB1	Transcription factor	4q24	rs4648022	4:102575280	Intron	Enhancer (USF1)	TF + Histone markers	0.64 (0.47-0.88)<br>0.81 (0.67-0.99)	540/532<br>1105/4460	lymphoma	(Nordmark *et al.*, 2011)<br>(Nordmark *et al.*, 2013)
OR2B11 (NLRP3)	cryopyrin	1q44	rs10732302	1:247458926	Intergenic	Unknown	DNase peak	1.43 (1.11-1.83)	391		(Nordmark *et al.*, 2011)
PKN1	Protein kinase C	19p13.12	rs3786654	19:14447009	Intron	Promoter	Histone markers	1.3 (1.0–1.7)	193/376		(Norheim *et al.*, 2014)
PKN1	Protein kinase C	19p13.12	rs10416904	19:14468001	Intron	Enhancer + Insulator (CTCF) + target of NMD	TF + DNase peak + histone markers	1.6 (1.1–2.5)	193/376		(Norheim *et al.*, 2014)
PKN1	Protein kinase C	19p13.12	rs2241362	19:14472960	Downstream	Promoter (Pol2, Taf1)	TF + DNase peak	0.9 (0.7–1.3)	193/376		(Norheim *et al.*, 2014)
PTPN22		1p13.2	rs2476601	1:113834846	Exon	Enhancer (STAT3) + Misense	TF + DNase peak/footprint	2.42 (1.24-4.75)	70/308	RA, DID, SLE, AITD, Addison	(Gomez *et al.*, 2005)
SLC25A40	Mitochondrial Carrier	7q21.12	rs10276819	7:87852190	Intron	Enhancer + target of NMD	No	0.5 (0.2–0.8)	193/376		(Norheim *et al.*, 2014)
STAT1	Transcription factor	2q32.2	rs13005843	2:190989900	Intron	Promoter (Pol2) + target of NMD	TF	0.55 (0.37-0.82)	391		(Nordmark *et al.*, 2011)
STAT4	Transcription factor	2q 32.2	rs10168266	2:191071078	Intron	Enhancer	Histone markers	1.44 (1.32-1.57)	542/1050	SLE, PBC, IBD, SSc	(Li *et al.*, 2013)
STAT4	Transcription factor	2q 32.2	rs7574865	2:191099907	Intron	Unknown	No	1.46 (1.09-1.97)<br>1.40 (1.21-1.62)<br>1.32 (1.01-1.73)	124/1143	SLE, PBC, SSc, IBD	(Korman *et al.*, 2008)<br>(Palomino-Morales *et al.*, 2010)<br>(Zheng *et al.*, 2013)
STAT4	Transcription factor	2q 32.2	rs10181656	2:191105153	Intron	Insulator (CTCF)	TF + DNase peak		368/711	CBP, IBD	(Nordmark *et al.*, 2009)
STAT4	Transcription factor	2q 32.2	rs7582694	2:191105394	Intron	Enhancer (Fos, Esr1)	TF + DNase peak	1.41 (1.14-1.73)<br>1.40 (1.15-1.70)<br>1.57 (1.27-1.93)	368/711<br>540/532<br>186/152	SLE, RA, SSc	(Nordmark *et al.*, 2009)<br>(Nordmark *et al.*, 2011)
TNF	Cytokine	6p21.33	rs1799964	6:31574531	Downstream	Promoteur (Pol2) + Insulator (CTCF)	TF + DNase peak	2.83 (0.27-7.8)	65/58	CD	(Gestermann *et al.*, 2010; Cay *et al.*, 2012)
TNF	Cytokine	6p21.33	rs1800629	6:31575254	Upstream	Enhancer (Egr1, cMyc)	eQTL + TF + DNase peak +	2.00 (1.61-2.49)<br>2.9	527/532<br>67/430	SLE, RA, AITD, IBD,	(Bolstad *et al.*, 2012)<br>(Correa *et al.*, 2005)

							SP1 motif			AIH, DID, cancer, allergy, lymphoma B, infection,	
TNFA1P3	Nf Kappa B signalling	6q23	rs5029939	6:137874586	Intron	Promoter (Pol2, IKZF1)	TF + DNase peak	1.67 (1.40-1.99)<br>1.28 (0.99-1.65)	542/1050<br>1105/4460	Polymyosite, SLE, SSc	(Li *et al.*, 2013)<br>(Nordmark *et al.*, 2013)
TNFA1P3	Nf Kappa B signalling	6q23	rs2230926	6:137874929	Exon	Promoter (Pol2) + Misense	TF + DNase peak	3.36<br>1.30 1.01-1.68)	574/451<br>1105/4460	SLE, RA, Lymphoma B	(Li, Zhang et al. 2013) (Nocturne *et al.*, 2013)<br>(Nordmark *et al.*, 2013)
TNFSF13B (BAFF)	B cell activating factor	13q33.3	rs9514828	13:108269025	Upstream	Enhancer (IRF4)	TF + DNase peak	0.23 (0.08-0.71)	111/137	Lymphoma B	(Nezos *et al.*, 2014)
TNFSF13B (BAFF)	B cell activating factor	13q33.3	rs12583006	13:108285104	Intron	Enhancer	Histone markers	0.52 (0.2-1.31)	111/137	Lymphoma B	(Nezos *et al.*, 2014)
TNFSF4 (CD252)	OX40 ligand	1q25.1	rs1234313	1:173197108	Intron	Enhancer	Histone markers		250/393	SLE, allergy, cancer	(Kong *et al.*, 2013)
TNFSF4 (CD252)	OX40 ligand	1q25.1	rs2205960	1:173222336	Upstream	Enhancer (NFKB1, MEF2A)	TF + DNase peak		250/393	SLE, SSc, PBC	(Kong *et al.*, 2013)
TNFSF4 (CD252)	OX40 ligand	1q25.1	rs1234314	1:173208253	Upstream	Enhancer	DNase peak		540/532	SLE, SSc,	(Nordmark *et al.*, 2011)
TNFSF4 (CD252)	OX40 ligand	1q25.1	rs1234315	1:173209324	Upstream	Enhancer	DNase peak	1.34 (1.14-1.64)	540/532	SLE, SSc, allergy, caner	(Nordmark *et al.*, 2011)
TNFSF4 (CD252)	OX40 ligand	1q25.1	rs2205960	1:173222336	Upstream	Enhancer (NFKB1, BATF)	Histone markers	1.11 (0.93-1.34)	555/597	SLE, Breast cancer	(Sun *et al.*, 2013a)
TNIP1	NF kappa B signaling	5q33.1	rs6579837	5:151055333	Intron	Enhancer + target of NMD	DNase peak	1.43 (1.20-1.71)<br>2.1	395/1975<br>540/532	-	(Lessard *et al.*, 2013)<br>(Nordmark *et al.*, 2011)
TNIP1	NF kappa B signaling	5q33.1	rs12109187	5:151069300	Intron	Enhancer	TF + Histone markers	1.43 (1.10-1.87)	1105/4460		(Nordmark *et al.*, 2013)
TNIP1	NF kappa B signaling	5q33.1	rs3792783	5:151076171	Intron	Promoter (Pol2)	TF + DNase peak	1.33 (1.16-1.52)	1105/4460	SLE, SSc	(Nordmark *et al.*, 2013)
TNIP1	NF kappa B signaling	5q33.1	rs7708392	5:151077924	Intron	Enhancer (WRNIP1)	TF + Histone markers	1.21 (1.08-1.36)	1105/4460	SLE, Behcet	(Nordmark *et al.*, 2013)
TNPO3		7q32.1	rs13246321	7:129061277	Downstream	Enhancer	Histone markers	1.70 (1.35-2.13)	540/532	-	(Nordmark *et al.*, 2011)
Trim21	Ro52 autoantigen	11p15.4	rs2855142	11:4389472	Intron	Enhancer	Histone markers + HEB motif		97/72	-	(Nakken *et al.*, 2001)

Abbreviations: SLE : systemic lupus erythematosus, RA : rheumatoid arthritis, SSc : systemic sclerosis ; IBD : inflammatory bowel disease, AITD : autoimmune thyroidis disease, DID : insulin-dependent diabetes, PBC : primary biliary cirrhosis, autoimmune hepatitis ; CD :celiac disease, MS : multiple sclerosis. *pSS patients with germinal centers versus pSS patients without germinal centers.
